# Compactness Determines the Success of Cube and Octahedron Self-Assembly

**DOI:** 10.1371/journal.pone.0004451

**Published:** 2009-02-12

**Authors:** Anum Azam, Timothy G. Leong, Aasiyeh M. Zarafshar, David H. Gracias

**Affiliations:** 1 Department of Biomedical Engineering, The Johns Hopkins University, Baltimore, Maryland, United States of America; 2 Department of Chemical and Biomolecular Engineering, The Johns Hopkins University, Maryland, United States of America; 3 Department of Chemistry, The Johns Hopkins University, Baltimore, Maryland, United States of America; University of East Piedmont, Italy

## Abstract

Nature utilizes self-assembly to fabricate structures on length scales ranging from the atomic to the macro scale. Self-assembly has emerged as a paradigm in engineering that enables the highly parallel fabrication of complex, and often three-dimensional, structures from basic building blocks. Although there have been several demonstrations of this self-assembly fabrication process, rules that govern *a priori* design, yield and defect tolerance remain unknown. In this paper, we have designed the first model experimental system for systematically analyzing the influence of geometry on the self-assembly of 200 and 500 µm cubes and octahedra from tethered, multi-component, two-dimensional (2D) nets. We examined the self-assembly of all eleven 2D nets that can fold into cubes and octahedra, and we observed striking correlations between the compactness of the nets and the success of the assembly. Two measures of compactness were used for the nets: the number of vertex or topological connections and the radius of gyration. The success of the self-assembly process was determined by measuring the yield and classifying the defects. Our observation of increased self-assembly success with decreased radius of gyration and increased topological connectivity resembles theoretical models that describe the role of compactness in protein folding. Because of the differences in size and scale between our system and the protein folding system, we postulate that this hypothesis may be more universal to self-assembling systems in general. Apart from being intellectually intriguing, the findings could enable the assembly of more complicated polyhedral structures (e.g. dodecahedra) by allowing *a priori* selection of a net that might self-assemble with high yields.

## Introduction

Nature utilizes self-assembly to fabricate structures on a wide range of size scales from the angstrom to the kilometer [1]. Recently, several researchers have attempted to mimic natural self-assembly by engineering components that interact and assemble into three-dimensional (3D) structures [1]–[3]. However, while natural self-assembly occurs with high fidelity, engineering principles guiding *a priori* design of complex structures with high defect tolerance are not well-understood.

One important natural self-assembling system that has been extensively studied is protein folding. In protein folding, it has been postulated that the net interactions of the building blocks need to result in a funnel-shaped potential energy landscape with minimal kinetic trapping [4]. However, exactly how to design a system featuring such minima is not entirely clear. Therefore, aside from relatively simple and unrealistic peptide sequences, the final structure of a folded protein is still extremely challenging to design or predict from its linear amino acid sequence [5], [6]. Several researchers have invoked the concept of compactness and a zipping-and-assembly model to elucidate protein folding [7]–[10]. The model states that with increasing compactness there are fewer accessible conformations during folding and that a small radius of gyration increases the likelihood of achieving the desired secondary structure in the protein [11]. While it is possible to explore this hypothesis using simulations, it is challenging to verify it experimentally. Additionally, it is not clear if such hypotheses are applicable to other self-assembling systems.

**Figure 1:**
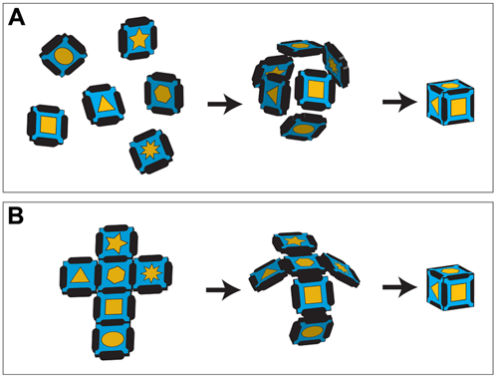
Schematic diagram showing the self-assembly of a cube from (A) six untethered panels and (B) six tethered panels. Since the number of conformations is greatly restricted by tethering as in (B), self-assembly occurs with much higher yield.

**Figure 2:**
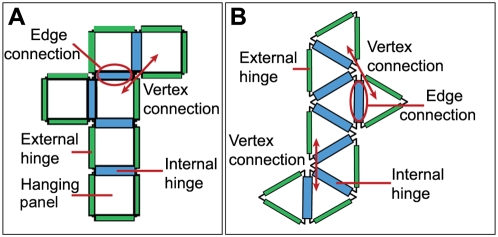
Schematic diagram of the net geometry. The diagram shows the (A) cube and (B) octahedron net geometry and illustrates the different kinds of topological connections and hinges.

Our group has focused on studying the self-assembly of two-dimensional (2D), patterned templates into micropolyhedra [12]. From an engineering perspective, patterned micropolyhedra may seem like simple objects, but it should be noted that self-assembly is the only strategy that has been demonstrated so far to fabricate such three-dimensionally patterned objects in a highly parallel manner. In order to accomplish self-assembly of a patterned polyhedron such as a cube, one might envision starting with six patterned, square panels that have mating edges (Fig. 1A). However, the number of configurations in which the six square panels can interact and join together is large, and this assembly results in too many kinetic minima or defect states to successfully result in the formation of a cube with high yield. Thus, we limited the number of configurations by tethering the square panels together in the form of a 2D net (Fig. 1B). Additionally, we utilized two types of hinges: internal hinges between panels and external hinges at the outer edges of each panel (Fig. 2); the hinges were composed of solder. Assembly occurred when the solder was liquefied and minimized its exposed liquid surface area. The surface tension caused the internal hinges to bead up, creating a torque that rotated the panels, and enabled the external hinges on adjacent panels to fuse when the panels met. Although most previous self-folding work has only utilized internal hinges, the addition of external hinges to self-folding structures fabricated in our research group has resulted in increased defect tolerance and self-correction; this has translated into high yield assembly of micropolyhedra.

It is known that not all arrangements of six square panels connected edge-to-edge will fold into a cube. If one is given a simple polygon (and its interior) in the plane, Alexandrov's theorem gives conditions under which this polygon can be folded by the identification of points of the polygon's boundary to a convex polyhedron or a double covering of a convex polygon [13]. Here, the full power of this theorem is not required. What will be considered instead are polygons that have fold lines (we call these lines internal hinges, which separate the original polygon's interior into polygonal panels) which will form convex polyhedra (with the panels becoming faces of the completed polyhedron) when folded along the fold lines and the edges of the polygonal boundary are joined together (Fig. 2). The term “net” is often used to describe this situation. Note that for some nets, when using the existing fold lines, it is possible to make either a non-convex polyhedron or a convex polyhedron depending on how the polygonal edges are joined together, e.g. the nets of the octahedron can form non-convex and regular octahedra. However, this does not arise for the cube. There are 11 nets that fold into a cube [14] and 11 that fold into octahedra, but the number of nets varies for different polyhedra. For example, the tetrahedron has two nets and the regular dodecahedron has 43380 nets [15]. The basic constraints in folding the polyhedral net are that the material must exhibit continuous folding, conserve distances along its surface and not self-intersect [16].

**Figure 3:**
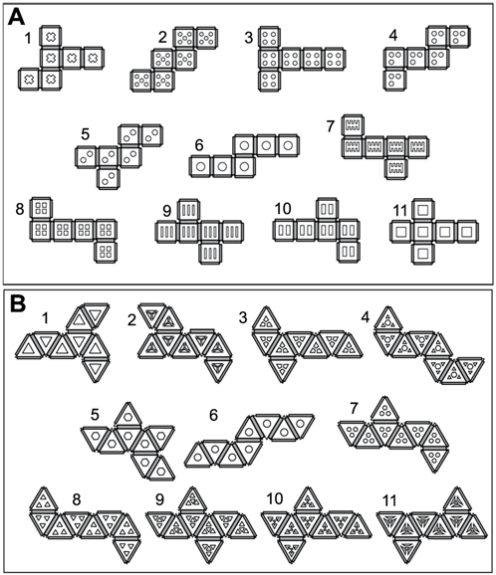
Schematic diagram of all the eleven 2D (A) cube and (B) octahedron nets.

When we first started assembling polyhedra, no design rules existed for which of the 11 nets would self-assemble with the highest yields. We picked the mirror-symmetric cruciform (net 11 in Fig. 3A) due to its familiarity, and it is used by several other groups [17]–[20]. In this paper, we systematically investigated the self-assembly of all 11 cube nets. We also investigated the self-assembly of the 11 octahedron nets, since the regular octahedron is the dual polyhedron for a cube; a dual polyhedron is one in which the roles of faces and vertices are interchanged when compared with the original polyhedron [21]. We recorded the number and types of defects observed during each assembly over 68 trials for each polyhedron. Although we observed that each net could fold into a well-formed polyhedron, a clear trend emerged for the number of defects in the assembly among the different nets. We observed that the cruciform net actually did not provide the best yields for assembling a cube. Also, there was a strong correlation between the success of each net folding into the desired polyhedron and purely geometric compactness factors, such as the nature of the connectivity of the different panels in the net design and a radius of gyration function.

## Results and Discussion

We used a previously established procedure for fabricating the 200 and 500 µm cubes and octahedra on silicon wafers [12] (see **Materials and Methods**); cube nets were processed across two wafers, while all of the octahedron nets were processed on one wafer. Each net was fabricated with nickel panels (square-shaped for cubes and equilateral-triangular shaped for octahedra) connected edgewise by solder hinges. The edges of each panel featured hinges; internal hinges (along fold lines) connected two panels, while external hinges were at the edges of the panels and did not connect to other panels. Each panel measured either 200 or 500 µm on each side, and adjacent panels were spaced apart by a width equal to 10% of the panel edge length. We electrodeposited solder at the panel edges to form the hinges, released the nets from the substrate and heated the structures until they folded at the hinges to form polyhedra. The samples on each wafer were constructed in close proximity to minimize any variations in the dimensions during lithographic processing. The wafers were organized such that a row of 11 nets was repeated multiple times. Each net featured a characteristic pattern on all panels to distinguish the polyhedra. Such an identification system was necessary, since cubes and octahedra resulting from different nets were assembled simultaneously to minimize any other process variations. It should be noted that at sub-mm size scales, the role of gravity in this self-assembling process is minimal [12]. Nevertheless, special care was taken in the design so that all of the panels on all nets had the same mass. Following a lift-off process from the substrate, the various nets were sorted, placed in random orientations in a dish and heated until surface tension forces drove them to fold into polyhedra. We folded the nets in batches, such that representatives of each were present. We defined the self-assembly of all the polyhedra in a dish as one trial and completed a total of 68 trials each for the 200 µm cubes and the octahedra. We also performed 36 trials each for 500 µm cubes and octahedra and observed that the folding trends (discussed later) were similar.

**Figure 4:**
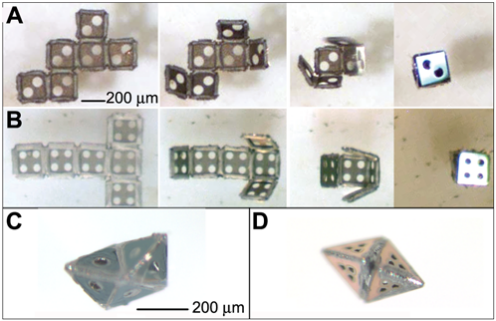
Cube folding dynamics and octahedral conformations. Two distinct folding dynamics during self-assembly were observed for cube nets: (A) net 5 follows pathway 1 and (B) net 3 follows pathway 2. Pathway 1 was characterized by independent folding of two clearly distinguishable sections of the net, which came together when the central hinge folded. Nets following pathway 2 have different folding rates for different sections of the net. Octahedron nets can fold into (C) non-convex boat-shaped or (D) regular octahedra.

For the cubes, we observed that each of the 11 nets folded by one of two distinct pathways (Fig. 4 A–B). The first pathway involved two clearly distinguishable sections of the net folding independently at equal rates and then coming together when a central hinge folded. The second folding pathway was characterized by different folding rates within the sections of the net. Nets 2, 4, 5, 7, 8, and 9 (Fig. 3) followed the first pathway; the remaining nets followed the second pathway. Fig. S1 in the Supporting Information section shows snapshots of all the 11 cube nets during folding. Interestingly, folding of octahedra appeared to follow more complicated pathways, and there were two possible final conformations, either the non-convex boat-shaped octahedron or the convex regular octahedron (Fig. 4 C–D). The formation of non-convex and regular octahedra depended both on the type of net as well as the folding sequence of the individual panels during assembly; some nets formed both types of octahedra.

**Figure 5:**
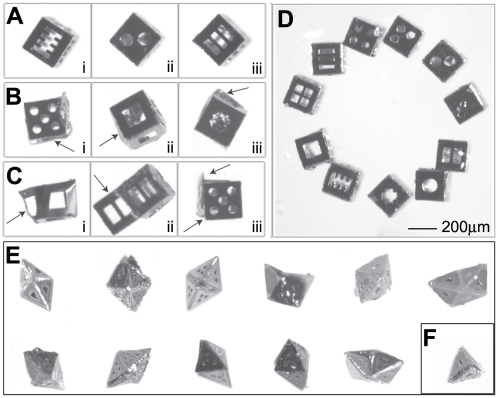
(A–C) Cubes and octahedra were classified according to the following criteria. (Ai–iii) “A” cubes have no defects. (Bi, Biii) “B” cubes may have one misaligned face, or display slight underfolding or overfolding. (Ci–iii) “C” cubes are (Ci) severely twisted, (Cii) have a missing or unfolded face, or (Ciii) have a severely misfolded/misaligned face. (D) All 11 cube nets were capable of folding into “A” cubes. (E) All 11 octahedron nets were also capable of all self-assembling into “A” octahedra. There are two conformations of the folding of the octahedron nets: the regular octahedron and the non-convex octahedron (boat shape). A common defect observed in the folding of octahedron nets was (F) a tetrahedron. All of these are 200-micron scale structures.

**Figure 6:**
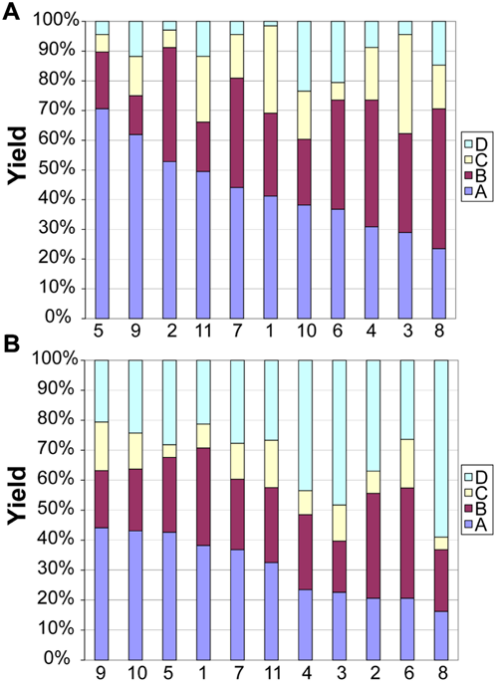
Distribution of defects in order of decreasing yield of “A” category (A) cubes and (B) octahedra. Violet denotes “A” category polyhedra; maroon denotes “B” category polyhedra; yellow denotes “C” category polyhedra; and light blue denotes “D” category polyhedra.

The data gathered from the assembly of 200 µm and 500 µm polyhedra indicated that all of the nets, with varying levels of defects (Fig. 5A–C), were capable of forming perfectly-folded polyhedra (Fig. 5 D–E). We organized the self-assembled cubes and octahedra into four categories (labeled A through D) according to their defects. We could not discern any defects in “A” polyhedra using optical microscopy. They had well-aligned faces and hinges that folded for form dihedral angles of 90° for cubes (Fig. 5A) and 109.4° for octahedra. “B” polyhedra were observed to have either one misaligned face (Fig. 4Bi, 4Biii) or slightly (deviation<15°) under/overfolded faces. Underfolding occurred when excess solder was present at a hinge between two faces, and overfolding occurred when an inadequate amount of solder was present in the hinge. “C” polyhedra were missing one face, or were severely (deviation>15°) over/underfolded (Fig. 5Cii, 5Ciii). In some cases with cubes, we observed a twist deformation and also classified those as “C” cubes (Fig. 5Ci). “D” polyhedra had two or more of the defects described for “C” polyhedra. Various other defects were observed in octahedra but not in cubes, which were a result of the comparatively more complicated folding mechanics; one common defect that occurred with the folding of octahedron nets was the overfolding of several sides, resulting in a tetrahedron (Fig. 5F) instead. Yields for cubes and octahedra are plotted in Figure 6 and listed in Tables S1, S2, with average ranges of “A” polyhedra plotted in Figure S2.

Five internal hinges along fold lines connect the six panels of each cube net; we refer to these connections as edge connections (Fig. 2A). Similarly, seven internal hinges are present along the fold lines and connect (through edge connections) the eight panels of each octahedron net (Fig. 2B). This method of identifying internal hinges along fold lines is attractive since it can be readily extended to the nets of other polyhedra. Vertex connections resemble topological connections described in protein folding models [7]. Vertex connections occur when panels are not directly connected to each other but are proximal and oriented at a specified angle to each other. There is one kind of vertex connection in the cube nets: when panels are located diagonally to each other, they share one vertex with an angle of 90° between the panels' exterior sides. There are two types of vertex connections in octahedron nets, as panels can be oriented with their exterior sides forming angles of 120° or 180° between them (Fig. 2B). A panel with no vertex connections to other panels in a cube is a hanging panel and is connected to the rest of the structure by only one edge connection. There are no hanging panels in octahedron nets, because each panel has at least one vertex connection. A more compact net results when each panel within the net has more vertex connections.

We also used the radius of gyration, another common parameter for determining compactness in protein structure, to quantify the compactness in the nets [10]. We defined the radius of gyration (*R_g_*) as 
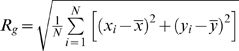
, where 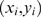 is the center of mass of each panel, 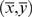 is the center of mass of the entire net, and N is the number of panels (see Tables S3, S4 in Supporting Information). We consider nets with a lower R_g_ as more compact.

**Figure 7:**
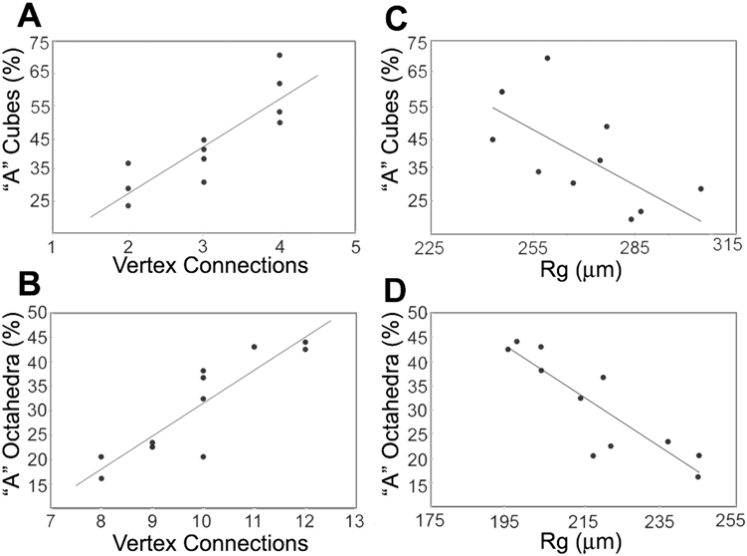
Trends of yield vs compactness. (A–B) Scatter plots of the percentages of “A” cubes and octahedra as a function of the number of vertex connections. (C–D): Scatter plots of the percentages of “A” cubes and octahedra as a function of R_g_. The trend lines have the following R-squared values. (A) y = 0.1478x−0.0219, R^2^ = 0.74; (B) y = 0.063x−0.2972, R^2^ = 0.74; (C) y = −0.0048x+1.7387, R^2^ = 0.49; (D) y = −0.0052x+1.4345, R^2^ = 0.77.

We observed strong correlations between the geometrical compactness of the 2D nets and the yields. Nets with more vertex connections and lower *R_g_* generated the most “A” polyhedra (Fig. 7). We performed statistical analysis under the assumption that the two factors were unrelated. Two-tailed *t*-tests were completed for statistical significance to verify that the vertex connections and *R_g_* correlated to yields of cubes and octahedra. Our statistical tests compared the percentages of “A” polyhedra to the corresponding values (per net) of vertex connections and *R_g_* for cubes and octahedra independently. The p-values fell within the 0.001% range dictated by the alpha value, which led us to conclude with 99.999% confidence that vertex connections and *R_g_* had statistical significance in average yields of different nets. The statistical significance of the unrelated factors further supports the hypothesis that net success in self-assembly is strongly driven by both of these geometrical factors.

Our experimental results can be rationalized as follows. We observed in both polyhedra that while panels folded first along edge connections, vertex connections enabled panels to lock together, thereby correcting for any errors in orientation. In fact, we observed that before folding together as a whole, nets would often undergo a period of solder readjustment and self-correction, in which panels moved into their lowest-energy positions. Thus, vertex connections enabled self-correction and enhanced defect tolerance. We also observed that hanging panels introduced defects in cube nets; however, hanging panels are not present in any octahedron net. Nets with hanging panels followed the second folding pathway, and the locking together of external hinges could not occur. Moreover, the hanging panel, connected only to one other panel by an internal hinge, needed to move a greater distance than the other panels in order for the cube to form. This extra movement also caused the side of the net with the hanging panel to fold more slowly than the other nets. The increase in motion of this hanging panel resulted in an increase in the error in the placement of the face and thus decreased contact between external hinges. Hence, cube nets with hanging panels tended to result in large numbers of “C” cubes.

Furthermore, our inclusion of *R_g_* as a factor for increasing yields of “A” cubes and octahedra is supported by various studies in biophysics. This function is related to the compactness of a structure and has been widely utilized in polymer and protein physics to quantify compactness of molecules [7]–[10]. These theoretical protein folding studies have shown that compactness in single polymer chains is a significant factor contributing to the internal folded protein structure (i.e. compact chains significantly increase secondary structure). It should be noted that several similarities and differences exist between our experimental study and the theoretical protein folding models. Our assembling polyhedra are similar to protein folding in the sense that both systems involve self-assembly and secondary interactions are important in both self-assembling processes. It is known that in the absence of secondary interactions between panels (i.e. in the absence of external hinges), the yield of our self-assembly is extremely low. However, it should be noted there are considerable differences in size scales, geometry and complexity between the two systems. Hence, while we believe this study may only offer limited insight on protein folding, it lends evidence to the concept that the principle of compactness may more universally impact the defect tolerance and yield of self-assembling systems.

In conclusion, we have experimentally uncovered a strong correlation between geometrical measures, namely compactness, and yield of self-assembly; additionally, the measures are simple and can be readily computed. Nevertheless, the reader should be cautioned that the measures utilized may not be unique. We did explore some other measures such as number and type of symmetry elements; however, they did not provide good correlations to yield. It should also be noted that it is extremely challenging to construct such model experimental systems in which the influence of these measures can be explored. However, these experimental studies are essential for gaining insight into the underlying process of self-assembly. Apart from being intellectually intriguing, the findings could also enable the assembly of more complicated structures (e.g. dodecahedra with 43380 nets) by allowing *a priori* selection of a single net that might self-assemble with high yields.

## Materials and Methods

### Fabrication details

We followed previously published fabrication procedures [22] with the differences that Shipley SC1827 photoresist [Rohm and Haas, www.rohmhaas.com] and 1∶6 diluted 351 Developer were used.

### Radius of gyration calculations

We used original net drawings in Autodesk AutoCAD to find the radii of gyration for all the cube and octahedron nets. The radius of gyration is the root-mean-square (rms) distance of the net's panels from the center of mass of the entire net region, and each net has an x-directional as well as a y-directional radius of gyration. To find the radii of gyration we first drew an outline around the shape, including hinge gaps, using the REGION command. The coordinates for the center of mass for this region was found using the MASSPROP command. We then found the center of mass of each panel. In cube nets this was done by drawing lines connecting the midpoints of sides opposite one another for each panel and finding where the lines intersected, and in octahedra nets we drew medians, connecting each vertex with the midpoint of the opposite side, and found the coordinates of their point of intersection. All nets were placed in the same coordinate plane, with their centers of mass placed at the origin. We then determined the x- and y- distances of the centroid of each panel to the centroid of the region in order to calculate the radius of gyration for the shape using the radius of gyration formula.

## Supporting Information

Table S1 Yields for all 200-micron cube nets
(0.05 MB DOC)

Table S2 Yields for all 200-micron octahedron nets
(0.05 MB DOC)

Table S3 R_g_ for all 200-micron cube nets
(0.03 MB DOC)

Table S4 R_g_ for all 200-micron octahedron nets
(0.03 MB DOC)

Figure S1 Video snapshots during folding of each of the cube nets. Two distinct folding dynamics were observed: nets 2, 4, 5, 7, 8 and 9 follow pathway 1 and the remaining nets follow pathway 2.
(9.77 MB TIF)

Figure S2 Average yield of “A” category (A) cubes and (B) octahedra over 12 wafer fragments. The values are ordered by decreasing percentage of “A” polyhedra and the standard deviation bars suggest the range of experimental variability.
(6.84 MB TIF)
