# Supplementary material for: Compactness Determines the Success of Cube and Octahedron Self-Assembly
Source: PLoS One. 2009 Feb 12;4(2):e4451. doi: 10.1371/journal.pone.0004451 (PMC2636878; doi:10.1371/journal.pone.0004451)
Supplement: Table S1 — Yields for all 200-micron cube nets (0.05 MB DOC) [file pone.0004451.s001.doc]

| **NET** | **%A** | **%B** | **%C** | **%D** |
| --- | --- | --- | --- | --- |
| **1** | 41.2% | 27.9% | 29.4% | 1.5% |
| **2** | 52.8% | 38.4% | 5.9% | 2.9% |
| **3** | 28.9% | 33.3% | 33.3% | 4.4% |
| **4** | 30.9% | 42.7% | 17.7% | 8.8% |
| **5** | 70.6% | 19.1% | 5.9% | 4.4% |
| **6** | 36.8% | 36.8% | 5.9% | 20.6% |
| **7** | 44.1% | 36.8% | 14.7% | 4.4% |
| **8** | 23.5% | 47.0% | 14.7% | 14.7% |
| **9** | 61.9% | 13.1% | 13.2% | 11.8% |
| **10** | 38.2% | 22.1% | 16.2% | 23.5% |
| **11** | 49.5% | 16.7% | 22.1% | 11.8% |
