# Supplementary material for: Compactness Determines the Success of Cube and Octahedron Self-Assembly
Source: PLoS One. 2009 Feb 12;4(2):e4451. doi: 10.1371/journal.pone.0004451 (PMC2636878; doi:10.1371/journal.pone.0004451)
Supplement: Table S2 — Yields for all 200-micron octahedron nets (0.05 MB DOC) [file pone.0004451.s002.doc]

| **NET** | **%A** | **%B** | **%C** | **%D** |
| --- | --- | --- | --- | --- |
| **1** | 38.2% | 32.5% | 8.0% | 21.3% |
| **2** | 20.6% | 35.0% | 7.4% | 37.0% |
| **3** | 22.6% | 17.1% | 12.0% | 48.3% |
| **4** | 23.5% | 25.0% | 8.0% | 43.5% |
| **5** | 42.6% | 25.0% | 4.2% | 28.2% |
| **6** | 20.6% | 36.8% | 16.2% | 26.4% |
| **7** | 36.8% | 23.5% | 12.0% | 27.7% |
| **8** | 16.2% | 20.6% | 4.2% | 59.0% |
| **9** | 44.1% | 19.1% | 16.2% | 20.6% |
| **10** | 43.1% | 20.6% | 12.0% | 24.3% |
| **11** | 32.5% | 25.0% | 15.8% | 26.7% |
