# Supplementary material for: Compactness Determines the Success of Cube and Octahedron Self-Assembly
Source: PLoS One. 2009 Feb 12;4(2):e4451. doi: 10.1371/journal.pone.0004451 (PMC2636878; doi:10.1371/journal.pone.0004451)
Supplement: Table S3 — Rg for all 200-micron cube nets (0.03 MB DOC) [file pone.0004451.s003.doc]

| **NET** | **R_g_ (μm)** |
| --- | --- |
| **1** | ­256.7 |
| **2** | 276.8 |
| **3** | 284.0 |
| **4** | 286.8 |
| **5** | 259.3 |
| **6** | 304.6 |
| **7** | 274.8 |
| **8** | 304.5 |
| **9** | 245.9 |
| **10** | 266.9 |
| **11** | 243.2 |
