# Supplementary material for: Compactness Determines the Success of Cube and Octahedron Self-Assembly
Source: PLoS One. 2009 Feb 12;4(2):e4451. doi: 10.1371/journal.pone.0004451 (PMC2636878; doi:10.1371/journal.pone.0004451)
Supplement: Table S4 — Rg for all 200-micron octahedron nets (0.03 MB DOC) [file pone.0004451.s004.doc]

| **NET** | **R_g_ (μm)** |
| --- | --- |
| **1** | 203.9 |
| **2** | 217.4 |
| **3** | 222.1 |
| **4** | 237.1 |
| **5** | 195.3 |
| **6** | 245.3 |
| **7** | 220.2 |
| **8** | 245.0 |
| **9** | 197.5 |
| **10** | 203.9 |
| **11** | 214.2 |
