# Supplementary figures and images for: Compactness Determines the Success of Cube and Octahedron Self-Assembly
Source: PLoS One. 2009 Feb 12;4(2):e4451. doi: 10.1371/journal.pone.0004451 (PMC2636878; doi:10.1371/journal.pone.0004451)

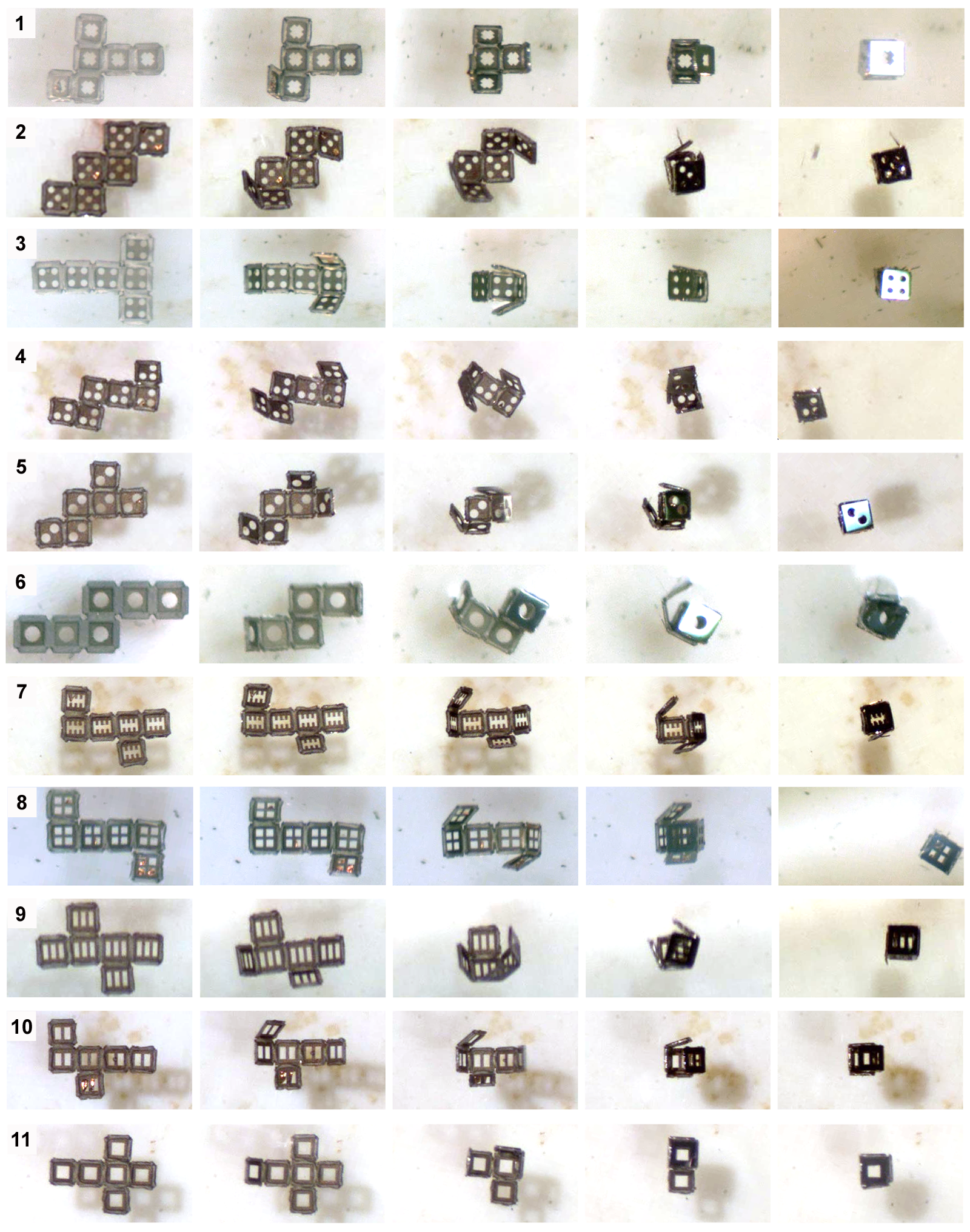

Supplement: Figure S1 — Video snapshots during folding of each of the cube nets. Two distinct folding dynamics were observed: nets 2, 4, 5, 7, 8 and 9 follow pathway 1 and the remaining nets follow pathway 2. (9.77 MB TIF) [file pone.0004451.s005.tif]

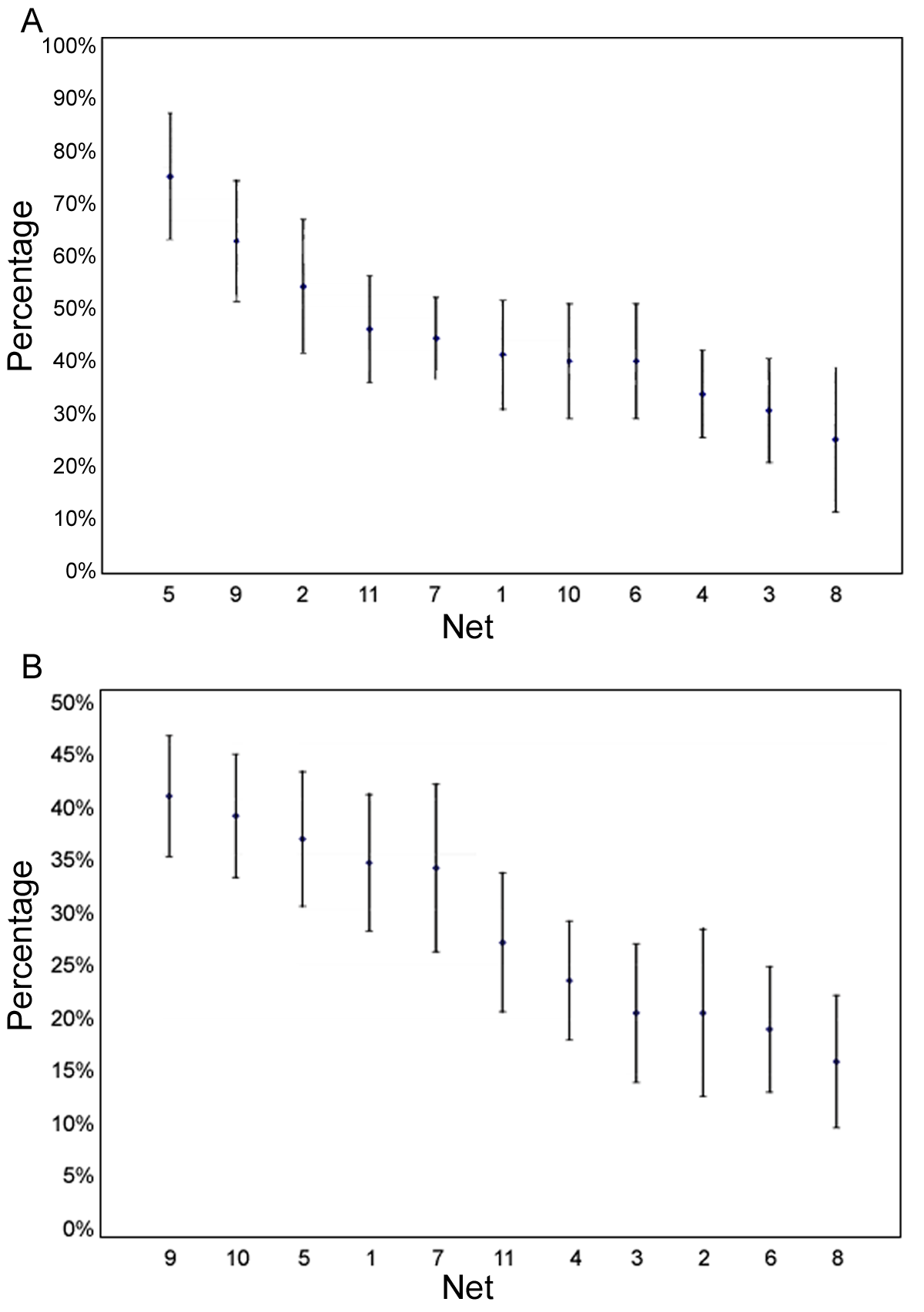

Supplement: Figure S2 — Average yield of “A” category (A) cubes and (B) octahedra over 12 wafer fragments. The values are ordered by decreasing percentage of “A” polyhedra and the standard deviation bars suggest the range of experimental variability. (6.84 MB TIF) [file pone.0004451.s006.tif]
